# Neuropsychological profile of CSF1R-related leukoencephalopathy

**DOI:** 10.3389/fneur.2023.1155387

**Published:** 2023-06-02

**Authors:** Beth K. Rush, Philip W. Tipton, Audrey Strongosky, Zbigniew K. Wszolek

**Affiliations:** ^1^Department of Psychiatry and Psychology, Mayo Clinic, Jacksonville, FL, United States; ^2^Department of Neurology, Mayo Clinic, Jacksonville, FL, United States

**Keywords:** CSF1R-related leukoencephalopathy, cognitive impairment, neuropsychology, cognitive profile, dementia

## Abstract

**Introduction:**

The neuropsychological profile of CSF1R-related leukoencephalopathy (CRL) is undefined. This study defines the profile, contrasts it with that of other dementia syndromes, and highlights measures sensitive to cognitive impairment.

**Methods:**

We administered a standardized battery of neuropsychological tests to five consecutive CRL cases.

**Results:**

The neuropsychological profile of CRL reflects impaired general cognitive function, processing speed, executive function, speeded visual problem solving, verbal fluency, and self-reported depression and anxiety. Confrontation naming and memory are preserved. Within cognitive domains, certain measures more frequently identified impairment than others.

**Discussion:**

CRL impairs general cognitive function, processing speed, executive function. Language and visual problem solving may be impaired if processing speed is required. Confrontation naming and memory are uniquely preserved, contrasting CRL to other dementia syndromes. Cognitive screens excluding processing speed and executive function may not detect CRL cognitive manifestations. Findings sharply define cognitive impairment of CRL and inform cognitive test selection.

## 1. Introduction

Adult-onset leukoencephalopathy with spheroids and pigmented glia (ALSP) is a neurologic disease characterized by rapidly progressive cognitive and motor impairment. It is a rare disease with few known cases; it is frequently under-or mis-diagnosed (1). Symptoms typically emerge in the fourth decade of life and there is a great deal of phenotypic heterogeneity even among individuals sharing the same pathogenic variant. It is a middle age disease with short survival. Multiple genes have been implicated in ALSP with the most common being the colony stimulating factor 1 receptor (CSF1R) gene and ALSP associated with CSF1R mutations have been subsequently termed CSF1R-related leukoencephalopathy (CRL) (2).

CRL is a primary microgliopathy and preferentially effects cerebral white matter (3). Pathological hallmarks include neuroaxonal spheroids, and pigmented macrophages that decrease in abundance as white matter degeneration advances (4). MRI is a useful tool to monitor disease progression and can demonstrate spread of white matter degeneration from focal to confluent distribution (5). MRI surveillance also demonstrates progressive cortical volume loss that often preferentially effects frontal and parietal lobes (6). The distribution of cortical atrophy influences individual phenotypes such that a patient may present very similarly to other primary neurodegenerative diseases, e.g., frontotemporal lobar degeneration (FTLD).

Cognitive impairment and dementia are well-recognized and referenced in descriptions of clinical phenotypes of CRL (7–9), with the most common description being that of “a frontal lobe syndrome” similar to behavioral variant frontal temporal dementia (bvFTD). There are no studies comparing the neuropsychological profile of CRL to those profiles of more common primary neurodegenerative diseases (e.g., FTLD clinical syndromes, Alzheimer’s disease, and Lewy body disease). General cognitive screening measures have been used to capture this cognitive impairment (10) but no studies document the neuropsychological profile of CRL or provide empirical support for which tests may best detect clinical impairment. In a case series of 3 siblings with the same *CSF1R* variant, “memory and frontal deficits” were identified on clinical evaluation, but specific tests are not mentioned (11). In a single case report, a neuropsychological test battery is documented but there are no other empirical studies published using this test battery in CRL (10). As cognitive decline is implicit in disease progression, it is imperative that objective, standardized, valid, and reliable cognitive measures are used for initial and subsequent neuropsychological evaluations for documenting cognitive trajectory over time (2). Knowing which measures are sensitive and specific to the neuropsychological impairment of CRL is important for designing clinical trials and evaluating treatment outcomes.

Herein, we present results from the initial clinical neuropsychological evaluation of five consecutive patients with genetically confirmed CRL. Patients underwent a standardized neuropsychological test battery evaluating domains of general cognitive function, attention, executive function, memory, visual spatial skill, processing speed, and self-reported emotional function. Subsequently, we describe the neuropsychological profile of symptomatic CRL and propose a standardized testing battery for the assessment of patients with confirmed or suspected CRL.

## 2. Materials and methods

Five consecutive patients were referred to a single neuropsychologist (BKR) for clinical neuropsychological evaluation after initial neurology evaluation (WSZ, PWT) confirmed a diagnosis of symptomatic CRL. Disease duration was based on years since earliest reported neurological symptoms including cognitive symptoms, personality/behavior symptoms, and/or motor symptoms.

### 2.1. Standard protocol approvals, registrations, and patient consents

The study protocol associated with data reported here was approved by the Mayo Clinic Institutional Review Board (FWA# FWA00005001) on July 17, 2020. The study was approved, via expedited review, as a minimal risk study. Study approval confirmed that the research met requirements for research with human participants in accordance with The Code of Ethics of the World Medical Association and 45 CFR 46 of the U.S. Department of Health & Human Services, Office for Human Research Protections. Written informed consent for research was obtained from all participants (or guardians of participants) in the study.

### 2.2. Neuropsychological assessment

A standardized clinical neuropsychological test battery was administered to each patient. The test battery prospectively included measures of general cognitive function and measures within the following specific cognitive domains: attention, processing speed, executive function, language, visual processing skills, memory, and self-reported emotional distress. Clinically relevant cut-off scores are empirically supported, published, and universally accepted for the cognitive screening measures (MMSE, MOCA) and emotional distress screening measures (BDI-II, BAI) and are described below. For all other neuropsychological measure scores, Z-scores were calculated for each patient based on the closest possible age-matched normative reference population. For example, a 58-year-old person’s score would be compared to a 60 year old normative score. Z-scores across tests within a cognitive domain were averaged for each patient and a radar chart was constructed to examine each patient’s neuropsychological profile of impairment across cognitive domains. A mean radar chart across the 5 patients was also constructed. To examine rates of impairment on cognitive tests within a domain, scores falling 1.5 standard deviations below the mean of an age-adjusted normative reference population for each measure were considered “clinically impaired.” For each test administered, the percentage of patients with an impaired score to examine whether specific cognitive tests with cognitive domains identified impairment.

#### 2.2.1. General cognitive function

The Folstein Mini Mental Status Exam (MMSE) (12), the Montreal Cognitive Assessment (MOCA) (13) and the Mattis Dementia Rating Scale—2 (DRS-2) (14) were administered. Scores less than 25 on the MMSE, less than 26 on the MOCA, and less than 124 on the DRS-2 identified impairment.

#### 2.2.2. Attention

The Attention subscale of the DRS-2 and the Digit Span subtest of the Wechsler Adult Intelligence Scale—IV (WAIS-IV) (15) assessed immediate attention, focused attention, and concentration.

#### 2.2.3. Processing speed

Part A of the original Trail Making Test (TMT) (16) or the Trail Making test of the Delis Kaplan Executive Functioning System (D-KEFS) (17), and Word Reading and Color Naming trials of the Stroop Test (18) or the DKEFS Color Word test were administered.

#### 2.2.4. Executive function

The Initiation and Conceptualization subtests of the DRS-2, Trail Making Test Part B or DKEFS Trails Condition 4, and the Color-Word trial of the Stroop or DKEFS Color Word test were administered.

#### 2.2.5. Language

The Boston Naming Test (BNT) (19), Controlled Oral Word Association (20) or DKEFS Letter Fluency, and Semantic Fluence or DKEFS Semantic Fluency were administered.

#### 2.2.6. Visual processing

The DRS-2 Construction subtest and WAIS-IV Block Design were administered.

#### 2.2.7. Memory

DRS-2 Memory subtest, California Verbal Learning Test—2 (CVLT-2) (21) learning over Trials, and Logical Memory I and Logical Memory II of the Wechsler Memory Scale—4 (WMS-IV) (22) were administered.

#### 2.2.8. Emotional distress

The Beck Depression Inventory—2 (BDI-II) (23) and the Beck Anxiety Inventory (BAI) (24) were administered. Scores >14 and > 7 on the BDI-II and BAI, respectively, identified impairment.

### 2.3. Comparison of neuropsychologic features in CRL to other dementia syndromes

Ratings compare the neuropsychological profile of CRL defined in this study to the most common presenting cognitive impairments in clinical phenotypes of other well-recognized dementia syndromes. A rating of (+++) is given to a cognitive symptom that is commonly accepted as a primary or even pathognomonic feature to the cognitive phenotype. A rating of (++) is ascribed to a cognitive symptom present to a moderate degree in the cognitive phenotype. A rating of (+) is ascribed to a cognitive symptom present to some or a mild degree in the cognitive phenotype. Finally, a (−) is ascribed to a cognitive symptom not common or present at all in the cognitive phenotype.

## 3. Results

**Figure 1:**
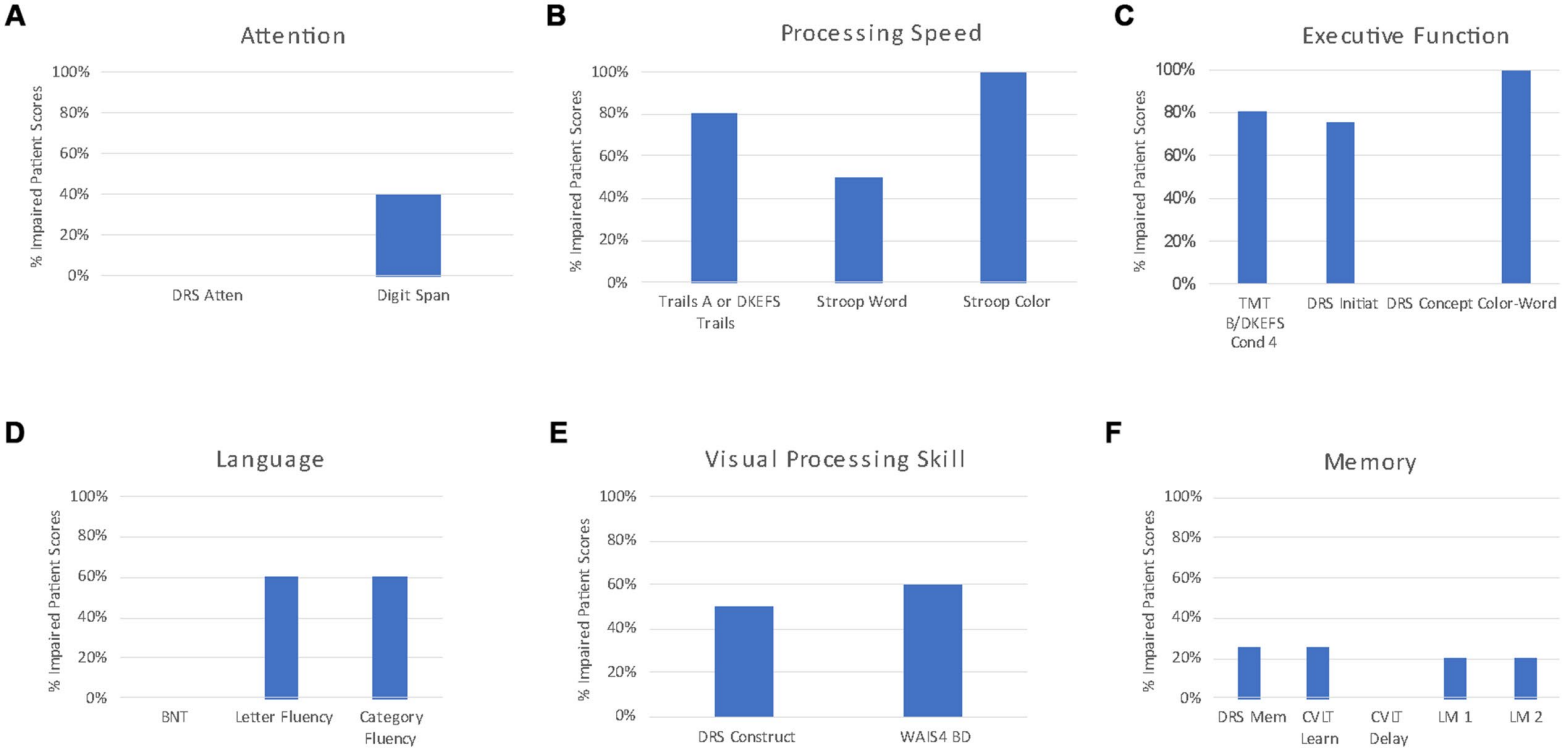
Radar plot of cognitive domain *Z*-scores for each patient and cohort mean. Concentric rings begin in the center at *z* = 0.0, with each expanding ring increasing by one standard deviation greater impairment to *z* = −2.0, and finally to *z* = −3.0. Colored lines represent individual patients. Thick black line represents mean across all patients.

**Figure 2:**
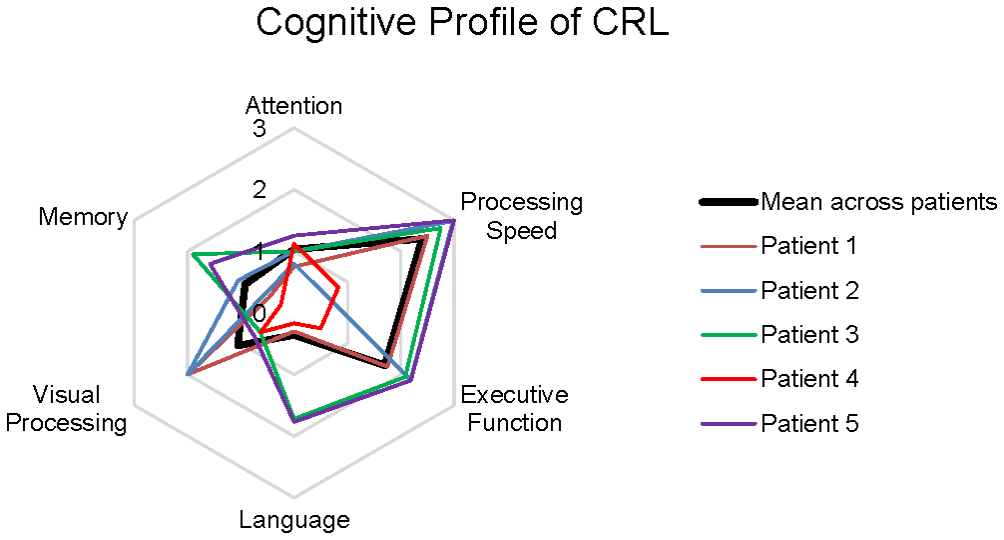
Bar graphs displaying percentage of impaired patient scores across tests within cognitive domains.

Patient descriptives are provided in Table 1. All 5 patients were Caucasian and right-hand dominant. Three patients identified as female and 2 as male, all between the ages of 37 and 51 years at the time of neuropsychological evaluation (mean: 42.2 years, standard deviaiton: 5.4 years). Patients ranged from 0.6 to 3.3 years of time between symptom onset and neuropsychological evaluation (mean: 2 years, standard deviation: 1 year). Raw test score descriptives for all neuropsychological measures administered are provided in Supplementary Table S1. For the General Cognitive Function domain, no patient’s score on the MMSE was impaired using the established cut-off score of <25. A single patient was administered the MOCA and had an impaired score of 19. DRS Total scores were impaired for three of four patients that completed the DRS [mean: 123.2, standard deviation: 4.6, median: 121.5, range (120–130)]. Normative z-scores for measures within each cognitive domain are averaged and plotted in Figure 1. The mean plot of z-scores by cognitive domain reveals that tests of processing speed and executive function are disproportionately impaired in CRL. Figure 2 illustrates the percentage of impaired scores captured by each neuropsychological test within a cognitive domain. For the Attention domain, no patients obtained an impaired general attention score (DRS-2 Attention) but 2 of 5 patients were impaired on the WAIS-IV Digit Span (Figure 2A). For the Processing Speed domain (Figure 2B), all patients had impaired scores on color naming speed but only half of the patients had impaired scores on word reading speed. Four of 5 patients were impaired in simple visual sequencing speed (Trail Making Test Part A or Conditions 1, 2, 3, and 5 of the DKEFS Trail Making test). For the Executive Function domain (Figure 2C), 4 of 5 patients had impaired scores in speeded mental flexibility (Trail Making Test Part B or Condition 4 of the DKEFS Trail Making Test) and 3 of 4 patients had impaired scores on test of inhibitory control (Stroop Color Word Test, Color-Word Trial or Trial 3 of the DKEFS Color Word Test). General initiation was impaired in 3 of 4 patients (DRS Initiation). Abstract verbal reasoning and simple reasoning was not impaired in any patients. For the Language domain (Figure 2D), no patients earned impaired scores in confrontation naming (BNT) but 3 of 5 patients had impaired scores in letter fluency and in category fluency. For the Visual Processing domain (Figure 2E), 2 of 4 patients had impaired scores on untimed visual constructional skill exercises (DRS Construction), and 3 of 5 patients had impaired scores on speeded visual constructional problem solving (WAIS-IV Block Design). For the Memory domain (Figure 2F), no patients had impaired learning efficiency or delayed recall on a multiple trial word list learning test (CVLT2). General immediate memory was impaired in 1 of 4 patients (DRS-2 Memory subtest) and immediate story memory and delayed story recall were impaired in 1 of the 5 patients assessed (Logical Memory). Median BDI-II and BAI scores were clinically significant across patients. On the BDI-II, scores ranged from 7 to 28 (mean: 17.2, standard deviation: 9.9). On the BAI, scores ranged from 15 to 18 (mean: 16.5, standard deviation: 2.1). Suicidal ideation on the BDI-II was endorsed at the time of evaluation in one of the five patients. Table 2 presents the neuropsychological profile of CSF1R-related leukoencephalopathy in contrast to those of other dementia syndromes associated with primary neurodegenerative disease.

**Table 1:** Patient demographics.

	*N* (%)	Mean	SD	Median	Range
Total	5	–	–	–	–
Age at time of testing (years)	–	42.2	5.4	41	(37–51)
Formal education (years)	–	16.6	3.1	16	(12–20)
Disease duration at time of testing (years)	–	2	1	2.2	(0.6–3.3)
Female	3 (60)	–	–	–	–
Right-handed	5 (100)	–	–	–	–
Caucasian	5 (100)	–	–	–	–

**Table 2:** Comparison of neuropsychologic features in CRL to other dementia syndromes.

	CRL	AD	LBD	FTD	PSP	CBS	Dep
General cognition	++	++	++	++	+	++	++
Attention	++	+	++	++	++	+	+
Processing speed	+++	+	+++	+	++	++	+++
Executive function	++	++	++	++	++	++	++
Naming	−	++	+	++	−	+	−
Verbal fluency	+++	++	++	+++	+	++	++
Visual processing	+	+	++	−	+	+	−
Learning/retrieval	−	+	++	++	+	+	++
Memory retention	−	+++	++	++	+	+	+
Emotional distress	+++	+	++	+	+	+	+++

CRL, CSF1R-related leukoencephalopathy; AD, Alzheimer’s dementia; LBD, lewy body dementia; FTD, frontal temporal dementia; PSP, progressive supranuclear palsy; CBS, corticobasal degeneration syndrome; Dep, depression/psychiatric Illness.

(−), negligible or absent; (+), present to mild degree; (++), present to moderate degree; (+++), present to very significant degree.

## 4. Discussion

A neuropsychological profile of CRL emerged from administering a standardized neuropsychological test battery to five consecutive patients referred for neuropsychological evaluation following confirmed diagnosis (Figure 1). Patients were impaired in general cognitive function to a degree that would suggest the presence of mild dementia. Processing speed and executive function were disproportionately impaired with additional cognitive inefficiencies observed in speeded visual processing and attention. In contrast, memory and language functions were relatively preserved. Patients self-reported clinically significant degrees of depression and anxiety symptoms. The emergent neuropsychological profile of CRL revealed reduced general cognitive function, slowed processing speed, impaired executive function, slowed word retrieval, slowed visual problem solving and self-reported symptoms of depression and anxiety. In contrast, reading, untimed naming, learning, and memory retention were relatively preserved. This finding is expected given that CRL preferentially affects white matter with cortical atrophy occurring as a later consequence of disease progression.

Within cognitive domains, some neuropsychological measures more frequently detected impairment relative to others. These differences further elucidate the specific neuropsychological profile of CRL and provide empirical support for test selection in describing cognitive manifestations of CRL. General cognitive function is impaired in CRL. Impairment was captured on the DRS-2 and MOCA, but not on the MMSE. The MMSE disproportionately emphasizes language and memory skills which are typically preserved in non-Alzheimer’s dementia syndromes such as Parkinson’s disease (PD). It has been previously shown that patients with PD may obtain normal MMSE scores despite scoring in the dementia range on other cognitive measures (25). In CRL, use of the MMSE may lead to false negative identification of cognitive impairment. Our results suggest that the DRS-2 Attention subtest may be less sensitive to impairment than a forward and backwards digit span task. In the evaluation of processing speed, measures of trail making and rates of word reading and color naming detect impairment frequently. Measures of mental flexibility and inhibitory control more commonly detected impairment than tests of abstract reasoning or simple reasoning. In the language domain, measures of speeded verbal fluency detected impairment, but a measure of untimed confrontation naming did not. In fact, there was no difference in the percentage of CRL patients impaired on letter fluency versus semantic fluency which is a pattern that emerges in other dementia syndromes (Table 2). This suggests that CRL patients may experience disturbances in language only to the degree that processing speed is inherent in the task. Alternatively, it is possible that impaired verbal fluency scores in CRL more likely related to impaired executive function than language function. Intersesting, disturbances in visual processing may only emerge when processing speed underlies performance as CRL patients were more frequently impaired on a speeded block assembly task than on untimed drawing tasks. In the memory domain, none of the neuropsychological measures frequently detected impairment in the cases. This contrasts to the prominent amnestic presentations observed in AD and MCI-Amnestic subtype cases and patterns of poor learning efficiency and memory retrieval observed in LBD, PSP, CBS, FTD, and depression cases. This suggests that memory measures may not need to be essential to neuropsychological test batteries designed to detect early cognitive impairment in CRL. More study is needed, with larger sample sizes and patients at varied stages of disease, to further inform which neuropsychological measures are most sensitive to the cognitive impact of CRL.

Table 2 compares the neuropsychological features of CRL with dementia syndromes of other primary neurodegenerative diseases. The neuropsychological profile of CRL is distinct from AD dementia or prodromal AD, i.e., amnestic mild cognitive impairment, in that memory is not impaired. Further, confrontation naming is often impaired in early AD as a function of proliferating temporal lobe cognitive systems dysfunction but remains preserved in CRL. The neuropsychological profile of CRL is also distinct from that of LBD. Both CRL and LBD share frontal subcortical cognitive systems compromise resulting in cognitive slowing, reduced attention and concentration, and diminished executive function. However, the neuropsychological profile of CRL does not involve frontal subcortical memory dysfunction and parietal–temporal-occipital junction visual systems dysfunction that is present in LBD. There are many similarities between neuropsychological presentations of CRL and FTD but generalized cognitive slowing is more pronounced and unique to CRL. PSP may be more likely to adversely impact learning, retrieval, and memory retention compared to CRL whereas general cognitive function may be more impaired in CRL relative to PSP. CRL and CBS neuropsychological profiles may be quite similar. This is not entirely unexpected as prior studies have documented overlap between clinical presentations of CBS and ALSP with confirmed CSF1R mutation (11, 26). Further research on distinguishing CRL and CBS neuropsychological profiles in early stage, or even prodromal disease, could ultimately be useful particularly in cases for which neuropsychological manifestations precede motor presentations. Although cognitive and behavioral changes experienced in CRL have been associated with behavioral variant FTD, to date, there have been no such studies directly comparing the neuropsychological profiles and such a study could be helpful in future research. Finally, the neuropsychological profiles of depression and CRL may be hard to distinguish as both involve impaired general cognitive function and prominent cognitive slowing. Our data suggest that learning, retrieval, and memory retention scores are more likely impaired in depression relative to CRL. Our data are the first to differentiate the neuropsychological profile of CRL from other primary neurodegenerative dementia syndromes and depression.

Patients with CRL reported mild depression symptoms and severe anxiety symptoms. Depression and anxiety are common in various forms of dementia and can even be observed in prodromal stages of dementia. For example, in a clinic-based sample of patients with Mild Cognitive Impairment, 40% of the sample reported significant depression (27). Rates of depression in atypical parkinsonian syndromes are more frequent and more severe than those reported in idiopathic PD (28). It is unknown if depression and anxiety are more prevalent in CRL than in other neurodegenerative conditions. From a methodological perspective, it is unclear whether self-report vs. informant-based neuropsychiatric symptom screening measures are most sensitive for screening neuropsychiatric symptoms in CRL. Prior work has suggested that the presence of diminished awareness, or anosognosia, accounts for variance in self-report accuracy when dementia patients must describe emotional distress relative to informant ratings (29). Prior descriptions of clinical symptoms in CRL have pointed out similarity to bvFTD but this study did not administer informant measures to evaluate frontal behavior and personality changes common in bvFTD and other associated frontal temporal lobar degeneration (FTLD) syndromes clinical syndromes. CRL symptom profiles on informant-based measures such as the Frontal Behavioral Inventory (FBI) (30) and the Neuropsychiatric Inventory—Questionnaire (NPI-Q) (31), which have been used in evaluating other FTLD syndromes, may be particularly interesting in CRL. Further research is needed to document mood, personality and behavior symptoms in CRL, in contrast to other FTLD clinical syndromes. Such work can elaborate on identify disease-specific neuropsychiatric features to CRL.

CRL is rare and it is challenging to report meaningful data on a series of consecutive cases and draw conclusions from small sample sizes. Ideally, all patients in this study would have received the exact same neuropsychological tests to assess each cognitive domain. Based on the availability of normative reference samples for raw score interpretation, patients received different versions of tests evaluating the same component of a given cognitive domain. This study did not correlate neuropsychological test performance with brain imaging findings, e.g., degree of corpus callosal atrophy, extent of white matter involvement (32), or the presence and extent of brain calcifications. Future correlative studies will improve understanding of the neuropsychological profile of CRL at various stages of disease and may be helpful in diagnostic decision-making algorithms by which to pursue interventions, symptom management strategies, or clinical trials. For example, if cognitive and imaging findings are sufficiently impaired, patients, families and clinicians may not decide an intervention offers the same yield verus a situation for which cognitive and imaging findings suggest a more nascent stage of the disease process.

This study is the first to document neuropsychological findings from a comprehensive test battery with a consecutive series of patients with CRL. Processing speed and executive functions are prominently impaired, but studies with larger patient cohorts and serial neuropsychological assessments will shed light on any dynamics of the CRL neuropsychological profile, which may change with disease progression. Deeper understanding of the CRL neuropsychological profile will strengthen counseling of patients and families and may guide treatment decisions.

## Data availability statement

The original contributions presented in the study are included in the article/Supplementary material, further inquiries can be directed to the corresponding author.

## Ethics statement

The studies involving human participants were reviewed and approved by the study protocol associated with data reported here was approved by the Mayo Clinic Institutional Review Board (FWA# FWA00005001) on July 17, 2020. The study was approved, via expedited review, as a minimal risk study. Study approval confirmed that the research met requirements for research with human participants in accordance with the Code of Ethics of the World Medical Association and 45 CFR 46 of the U.S. Department of Health and Human Services, Office for Human Research Protections. Written informed consent for research was obtained from all participants (or guardians of participants) in the study. The patients/participants provided their written informed consent to participate in this study.

## Author contributions

BR was the primary and corresponding author to this study. BR completed clinical evaluation of the patients, the data analyses, the primary preparation of the manuscript, and created supporting of tables and figures. PT was a co-author contributing to the clinical evaluation of the patients, data analyses, the creation of tables and figures, and preparing the manuscript. AS was a co-author and contributed to the clinical and research evaluation of the patients, assisted with data collection, and contributed to the analyses. ZW was a senior author contributing to the clinical and research evaluation of the patients, data analyses, and manuscript preparation. All authors contributed to the article and approved the submitted version.

## Conflict of interest

The authors declare that the research was conducted in the absence of any commercial or financial relationships that could be construed as a potential conflict of interest.

## Publisher’s note

All claims expressed in this article are solely those of the authors and do not necessarily represent those of their affiliated organizations, or those of the publisher, the editors and the reviewers. Any product that may be evaluated in this article, or claim that may be made by its manufacturer, is not guaranteed or endorsed by the publisher.

## Supplementary material

The Supplementary material for this article can be found online at: https://www.frontiersin.org/articles/10.3389/fneur.2023.1155387/full#supplementary-material
