# Supplementary material for: Neuropsychological profile of CSF1R-related leukoencephalopathy
Source: Front Neurol. 2023 Jun 2;14:1155387. doi: 10.3389/fneur.2023.1155387 (PMC10272847; doi:10.3389/fneur.2023.1155387)
Supplement: Supplementary file 1 [file Table_1.docx]

| Supplementary Table 1. Descriptive statistics for raw scores on neuropsychological measures. | | | | | |
| --- | --- | --- | --- | --- | --- |
| **Test Name** | **n** | **Mean** | **SD** | **Median** | **Range** |
| MMSE | 5 | 25.6 | 2.7 | 26 | (22 – 29) |
| MOCA | 1 | 19 | - | - | - |
| DRS Total | 4 | 123.3 | 4.6 | 121.5 | (120 – 130) |
| DRS Attention | 4 | 33.5 | 1.3 | 33 | (32 – 35) |
| DRS Initiation | 4 | 28 | 5 | 26.5 | (24 – 35) |
| DRS Construction | 4 | 5.3 | 1 | 5.5 | (4 – 6) |
| DRS Conceptualization | 4 | 35.3 | 1 | 35.5 | (34 – 36) |
| DRS Memory | 4 | 21.5 | 1.7 | 21 | (20 – 24) |
| Digit Span | 5 | 19.6 | 3.6 | 21 | (15 – 24) |
| Block Design | 5 | 21.2 | 9.8 | 20 | (10 – 36) |
| TMT, Part A | 3 | 81.3 | 49.6 | 77 | (34 – 133) |
| TMT, Part B | 3 | 225.3 | 131 | 301 | (74 – 301) |
| DKEFS Trails, 1 | 2 | 42 | 0 | 42 | 42 |
| DKEFS Trails, 2 | 2 | 91 | 15.6 | 91 | (80 – 102) |
| DKEFS Trails, 3 | 2 | 105.5 | 62.9 | 105.5 | (61 – 150) |
| DKEFS Trails, 4 | 2 | 173.5 | 94 | 173.5 | (107 – 240) |
| DKEFS Trails, 5 | 2 | 105.5 | 62.9 | 105.5 | (61 – 150) |
| Stroop Word | 2 | 62 | 34.6 | 62.5 | (38 – 87) |
| Stroop Color | 2 | 38 | 14.1 | 38 | (28 – 38) |
| Stroop CW | 2 | 21 | 9.9 | 21 | (14 – 28) |
| DKEFS Color | 2 | 63.5 | 14.8 | 63.5 | (53 – 74) |
| DKEFS Word | 2 | 34.5 | 3.5 | 34.5 | (32 – 37) |
| DKEFS CW | 2 | 98 | 25.4 | 98 | (80 – 116) |
| BNT | 4 | 56.5 | 3.4 | 57 | (52 – 60) |
| Letter Fluency | 2 | 21 | 5.7 | 21 | (17 – 25) |
| Semantic Fluency | 2 | 34.5 | 7.8 | 34.5 | (29 – 40) |
| DKEFS Letter Fluency | 3 | 20.3 | 24.2 | 10 | (3 – 48) |
| DKEFS Semantic Fluency | 3 | 19.7 | 9.8 | 14 | (14 – 31) |
| Logical Memory I | 5 | 22.4 | 4.8 | 22 | (15 – 28) |
| Logical Memory II | 5 | 16.8 | 8.6 | 19 | (2 – 24) |
| CVLT2 Learning over trials | 4 | 41.5 | 10.6 | 41 | (29 – 55) |
| CVLT2 Long Delay Free Recall | 4 | 9 | 3.4 | 9 | (6 – 12) |
| Beck Depression Inventory—2 | 5 | 17.2 | 9.9 | 15 | (7 – 28) |
| Beck Anxiety Inventory | 2 | 16.5 | 2.1 | 16.5 | (15 – 18) |
